# Xanthohumol microbiome and signature in adults with Crohn’s disease (the XMaS trial): a protocol for a phase II triple-masked, placebo-controlled clinical trial

**DOI:** 10.1186/s13063-022-06782-z

**Published:** 2022-10-22

**Authors:** Blake O. Langley, Jennifer Joan Ryan, John Phipps, Lita Buttolph, Brenna Bray, Joseph E. Aslan, Thomas O. Metz, Jan F. Stevens, Ryan Bradley

**Affiliations:** 1 Helfgott Research Institute, National University of Natural Medicine (NUNM), 2220 SW 1st Ave, Portland, OR 97201 USA; 2 School of Nursing, University of Washington, 1959 NE Pacific St, Seattle, WA 98195 USA; 3 School of Medicine, Oregon Health and Science University, 3303 S Bond Ave, Portland, OR 97239 USA; 4 Biological Sciences Division, Pacific Northwest National Laboratory, 902 Battelle Blvd, Richland, WA 99354 USA; 5 College of Pharmacy and the Linus Pauling Institute, Oregon State University, 1601 SW Jefferson Way, Corvallis, OR 97331 USA; 6 Herbert Wertheim School of Public Health and Human Longevity Science, University of California, San Diego, 9500 Gilman Dr, La Jolla, CA 92093 USA

**Keywords:** Natural product, Xanthohumol, Crohn’s disease, Inflammatory bowel disease, Microbiome, Inflammation, Safety, Tolerability, Randomized controlled trial

## Abstract

**Background:**

Xanthohumol (XN), a bioactive flavonoid from *Humulus lupulus* with anti-inflammatory properties, has potential benefits for patients with Crohn’s disease (CD), a type of inflammatory bowel disease. We recently completed and published results of a placebo-controlled phase I clinical trial demonstrating the safety and tolerability of 24 mg XN daily for 8 weeks. The present study aims to evaluate the safety and tolerability of the same dose of XN adults with clinically active CD in a placebo-controlled phase II clinical trial. Additional aims will assess the impact of XN on inflammatory biomarkers, platelet function, CD clinical activity, and stool microbial composition. The metabolism of XN will also be evaluated. This article provides a model protocol for consideration in investigations of XN or other natural products in disease states.

**Methods:**

A triple-masked, randomized, placebo-controlled trial will be conducted in adults with clinically active CD. Participants (*n* ≤ 32) will be randomized to either 24 mg encapsulated XN per day or placebo and followed for 8 weeks. Throughout the trial, participants will be queried for adverse events. Biomarkers of clinical safety, blood and stool markers of inflammation, platelet function, Crohn’s Disease Activity Index score, stool microbial composition, and XN metabolite profiles in blood, urine, and stool will be assessed every 2 weeks.

**Discussion:**

We describe the protocol for a phase II clinical trial that evaluates the safety and tolerability of XN in adults with active CD, as well as evaluate metabolism and mechanisms that are relevant to CD and other diseases with underlying inflammation and/or gut permeability. The effects of XN on inflammatory biomarkers, platelet function, the microbiota, and multi-omics biomarkers measured in this phase II trial of adults with CD will be compared to the effects of XN in healthy adults in our previous phase I trial. The results of the study will advance the evidence guiding the use of XN in patients with CD.

**Trial registration:**

ClinialTrials.gov NCT04590508. Registered on October 19, 2020

## Administrative information

Note: the numbers in curly brackets in this protocol refer to SPIRIT checklist item numbers. The order of the items has been modified to group similar items.

**Table:** 

Title {1}	Xanthohumol microbiome and signature in adults with Crohn’s disease (the XMaS trial): a protocol for a phase II triple-masked, placebo-controlled clinical trial
Trial registration {2a and 2b}	ClinicalTrials.gov (NCT04590508) and posted on October 19, 2020. All items per WHO trial registration requirements can be found within the body of the protocol.
Protocol version {3}	V1.13 approved August 31, 2021
Funding {4}	The XMaS Trial received funding from the National Center for Complementary and Integrative Medicine (NCCIH; R01 AT010271 and R01 AT010271-02S1) and National Heart, Lung, and Blood Institute (NHLBI; R01HL146549 and R01HL146549-02S1) of the National Institutes of Health. XanthoFlav >99% Pure XN raw material was provided by Hopsteiner, Inc. XN and placebo capsules were provided by Metagenics, Inc. Drs. Ryan, Phipps, and Bradley have previously received grant support and study product donations from Metagenics, Inc. which supported the phase II XMaS study by manufacturing the experimental drug used in this trial.
Author details {5a}	Blake O. Langley^1, 2^, Jennifer Joan Ryan^1^, Lita Buttolph^1^, John Phipps^1^, Janet K. Jansson^3^, Joseph E. Aslan^4^ , Thomas O. Metz^3^, Jan F. Stevens^5^, , Ryan Bradley^1, 6^ ^1^ National University of Natural Medicine, Helfgott Research Institute 2220 SW 1^st^ Ave, Portland, OR 97201 ^2^ University of Washington, School of Nursing 1959 NE Pacific St, Seattle, WA 98195 ^3^Pacific Northwest National Laboratory, Biological Sciences Division 902 Battelle Blvd, Richland, WA 99354 ^4^Oregon Health and Science University, School of Medicine 3303 S Bond Ave, Portland, OR 97239 ^5^ Oregon State University, College of Pharmacy and the Linus Pauling Institute 1601 SW Jefferson Way, Corvallis, OR 97331 ^6^University of California, Herbert Wertheim School of Public Health and Human Longevity Science 9500 Gilman Dr, La Jolla, CA 92093
Name and contact information for the trial sponsor {5b}	National University of Natural Medicine Contact: Ryan Bradley, ND, MPH rbradley@nunm.edu
Role of sponsor {5c}	This trial is an investigator initiated clinical trial sponsored by the National University of Natural Medicine (NUNM) and funded by the NCCIH of the NIH. The NIH provided regular feedback on the trial design, which was included in the trial protocol. Purified XN was provided by Hopsteiner, and the capsule formulation was manufactured by Metagenics, Inc. Neither the study funders nor the material suppliers were involved in the conduct of the trial, data analysis, or interpretation. No authors hold stock in Metagenics, Inc. or its parent company and have no financial ties to the outcome of the proposed clinical research.

## Introduction

### Background and rationale {6a}

Phased investigations of the safety, tolerability, and efficacy of natural products have posed unique challenges in the drug development model over the last decades [1, 2]. Stakeholders including the National Center for Complementary and Integrative Health (NCCIH) identify these investigations as a top priority in the coming years [3]. The aim of this article is to provide a protocol for a placebo-controlled phase II clinical trial investigating the safety and tolerability of a natural product in adults with Crohn’s disease (CD).

Xanthohumol (XN), derived from resins of the female cones of the hops plant (*Humulus lupulus*), is a potential natural drug early in the development pipeline. XN has demonstrated anti-inflammatory and antioxidant activity in vitro and in vivo [4, 5], prebiotic effects on the gut microbiome and the metabolome in vivo [6, 7], and upstream regulatory effects on gene expression, specifically on nuclear erythroid 2-related factor 2 (Nrf2), nuclear factor-κ B (NFκB), and the farnesoid-X receptor (FXR) [8–13]. Together, these mechanisms of action have the potential to broadly benefit patients with a variety of conditions, particularly those with excessive inflammation and/or elevated intestinal permeability. This is especially true in patients with CD, where measurements of circulating inflammatory cytokines, clinical activity indices such as the Crohn’s Disease Activity Index (CDAI), hematologic markers of organ function, and monitoring of adverse events throughout the interventional period are minimum requirements to meet recommendations for a comprehensive assessment of safety and tolerability, while also providing early surrogate indicators of potential efficacy [14].

Our recent phase I clinical trial demonstrated the safety and tolerability of 24 mg/day of XN over 8 weeks in healthy adults [15], corroborated by previous animal and human pharmacokinetics models indicating XN is safe to consume as an isolated constituent [16–19]. Furthermore, the US Food and Drug Administration (FDA) classifies both hops and hops oil as “Generally Recognized as Safe” (GRAS) [20]. To date, recurrent daily administration of XN has not yet been evaluated for safety or tolerability in a population with acute or chronic disease. Due to its documented in vitro and in vivo mechanisms related to inflammatory conditions like CD, it is desirable to determine the potential of XN as a natural drug candidate [4–13].

To further this goal, this protocol reports the design of a phase II trial in participants with clinically active CD. Upon enrollment, up to 32 participants will be randomized to take 24 mg XN encapsulated with a rice protein vehicle, or an encapsulated rice protein placebo, daily for 8 weeks. Hematologic biomarkers of safety, CDAI scores, inflammatory biomarkers, platelet function, gut microbiome composition, and XN metabolite profiles in plasma, urine, and stool will be monitored every 2 weeks to evaluate the safety and tolerability of XN in CD and provide a comparison in metabolism against samples previously collected in healthy adults.

### Objectives {7}

The main objective of the phase II trial is to determine the safety and tolerability of 24 mg/day of orally delivered XN compared to a placebo over 8 weeks in a group of adults with active CD. Secondary objectives of this study include assessing the impact of XN on the clinical activity of CD, inflammatory biomarkers that are often elevated in patients with CD, the composition of the gut microbiome in patients with CD compared to healthy adults, and the hemostatic function of platelets, as well as measuring the metabolic pathways and metabolite concentrations of XN. A summary of objectives and outcomes can be found in Table 1.

**Table 1:** Objectives and outcomes

Objectives Assessment of:	Measures	Method of assessment
*1. Safety and tolerability*	i. RBC, WBC, platelets ii. eGFR, electrolytes, BUN:Cr, AST, ALT, GGT, alkaline phosphatase, bilirubin iii. Heart rate, blood pressure, temperature, body mass index (BMI) iv. Adverse events and quality of life	i. Complete blood count (CBC) ii. Comprehensive metabolic panel (CMP) iii. Vital sign measurement iv. Adverse event monitoring and PROMIS-29 questionnaire
*2. Disease activity*	i. Clinical activity	i. Crohn’s Disease Activity Index (CDAI)
*3. Inflammation*	i. Inflammatory cytokines ii. Fecal calprotectin	i. Serum or plasma analysis ii. Fecal analysis
*4. Gut permeability and endotoxemia*	i. CD14, lipopolysaccharide (LPS) and LPS-binding protein, intestinal fatty acid binding protein	i. Plasma analysis
*5. Platelet function*	i. Platelet signaling, secretion, and procoagulant activity	i. Proteomic analysis ii. Flow cytometry
*6. Microbiome and bile acid compositions*	i. Metagenomic DNA sequencing and taxonomic profiling ii. Proteomic characterization iv. Bile acid profiling	i. Stool analysis ii. Activity-based proteomic assay iii. Plasma analysis
*7*. *Metabolic profile of XN*	iv. Xanthohumol (XN), isoxanthohumol, 6-prenylnaringenin, 8-prenylnaringenin, desmethylxanthohumol (DXN), dihydro-desmethylxanthohumol (DDXN)	iv. 24-h urinalysis, plasma analysis, stool analyses

#### Hypotheses

We hypothesize 24 mg/day of XN will be safe and tolerable in an adult population with active CD over an 8-week period. This will be supported by observing no greater than a 50% greater frequency in differences of laboratory abnormalities or adverse events in the XN group. We also hypothesize XN will improve the clinical activity and reduce inflammatory biomarker concentrations in CD, demonstrate a metabolite profile different than that of healthy adults, and generate a specific gut microbiome signature. These comparisons will be achieved by measuring data against that from the phase I clinical trial of the same interventional product and period.

### Trial design {8}

This study is a phase II, triple-blind, 1:1 drug:placebo allocation, parallel-arm, randomized, placebo-controlled trial under an investigational new drug (IND) application to the US FDA. The trial is registered with ClinicalTrials.gov (NCT04590508).

## Methods: participants, interventions, and outcomes

### Study setting {9}

Clinical visits and specimen preparation will be conducted at the National University of Natural Medicine (NUNM) Helfgott Research Institute in Portland, OR.

### Eligibility criteria {10}

**Fig. 1:**
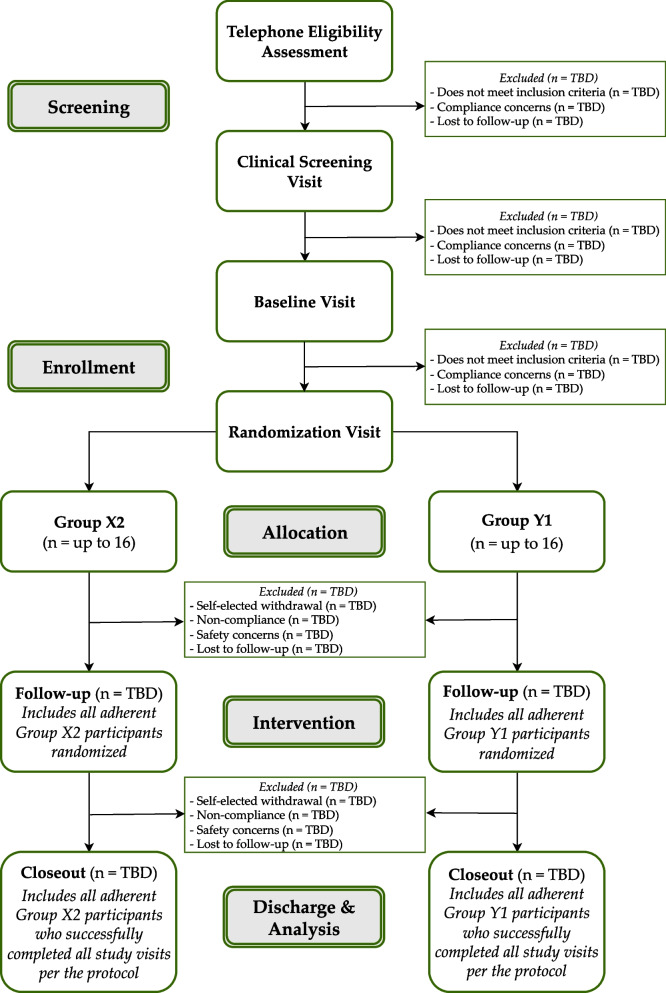
Flow of participation

Eligibility criteria are delineated in Table 2. Delineation of procedure flow and study visit descriptions can be found in Fig. 1 and Table 3, respectively. Candidates who meet modifiable exclusion criteria (e.g., taking prohibited dietary supplements) will be given the option to “wash out” for 14 days and re-contact the study team.

**Table 2:** Participant eligibility

Inclusion criteria	Exclusion criteria
• Adults 21–70 years of age • Active Crohn’s disease not in remission based on a CDAI score >150 • Willing to take isolated XN as a dietary supplement for 8 weeks • Willing to have blood drawn biweekly and fast for 10–12 h before blood draws • Willing and able to collect biweekly stool samples at home • Willing and able to collect a 24-h urine sample before each study visit • Able to speak, read, and understand English • Able to provide written informed consent • Non-smokers (including tobacco and Cannabis products in any delivery including cannabidiol) • For individuals of child-bearing potential, willing to use an intrauterine device or two other concurrent forms of birth control to prevent pregnancy while enrolled	• Variable dosing of anti-inflammatory medications >1×/week • Currently or recent (within last 14 days): o Taking any dietary supplements containing xanthohumol, flavonoids, or other “anti-inflammatories” including curcumin, turmeric, fenugreek, hops, rosemary, ginger, white willow, devil’s claw, fish oil (doses >1 g/day), or quercetin o Receiving intravenous nutrition support therapy o Taking anti-coagulant or anti-platelet prescription medications o Taking antibiotic, antiparasitic, or antifungal medications orally or intravenously o Initiated of or changes to supplements or medications o Initiated or changes to an exercise regimen o Initiated changes to a food plan o Involvement of a significant diet, weight loss, or very low-calorie liquid diet programs o Use of illicit drugs/substances o Participation in an interventional research study • Consumption of more than 1 beer per day or typical intake of more than 2 alcohol-containing beverages per day, more than 14 per week, or more than 4 in any single day within the past 14 days • Hospitalization (for any reason other than a scheduled medical procedure) within the last 3 months • Gastrointestinal surgery within the last 3 months • Malignancy within the last 5 years (except basal cell carcinoma, squamous cell carcinoma, and/or carcinoma in situ of the cervix) • Women who are lactating, pregnant, or planning pregnancy within the next 4 months • Do not have an active primary care provider or specialist (i.e., gastroenterologist) managing their CD

**Table 3:**
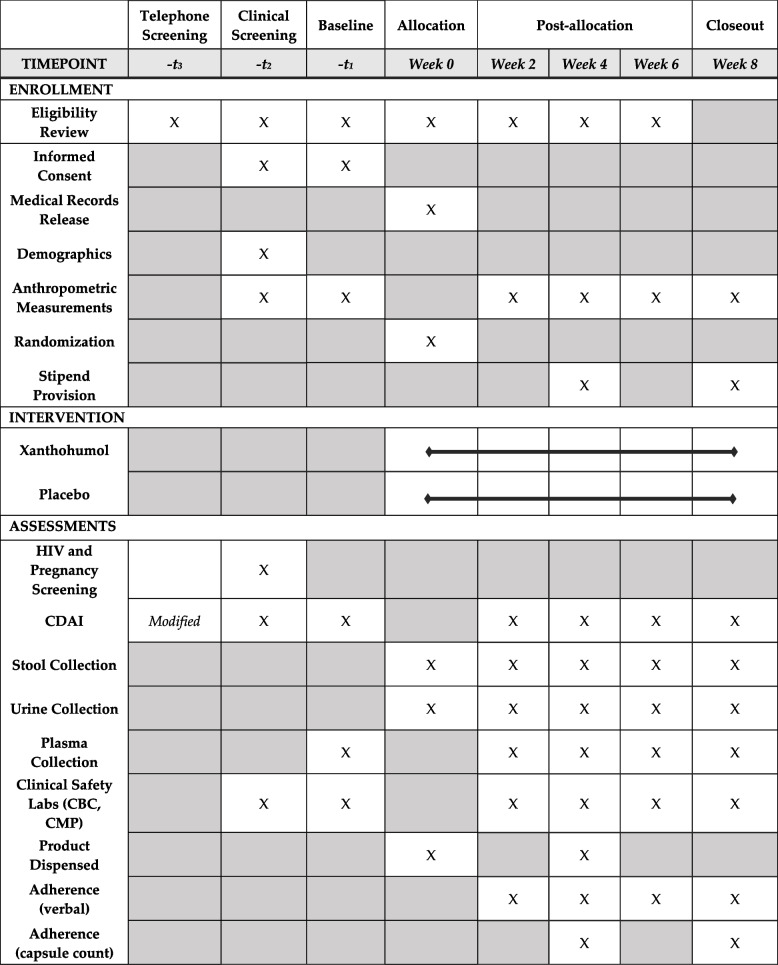
Study procedures

*CDAI* Crohn’s Disease Activity Index, *CBC* complete blood count, *CMP* comprehensive metabolic panel

### Who will take informed consent? {26a}

All possible efforts will be taken to maintain the privacy of participant information throughout the trial. All study staff will be trained in and maintain up-to-date certification in Good Clinical Practices, Human Subjects Research, the Responsible Conduct of Research, and HIPAA regulations. A member of the study staff will obtain informed consent at the screening and baseline visits prior to initiating any study procedures. Telephone screening will be conducted to evaluate participant eligibility per the criteria noted in Table 2 and are assigned an alpha-numeric screening ID. Participants who remain eligible following the phone screen will be invited to an in-person clinical screening visit. Informed consent for the remainder of the trial will be obtained at the baseline visit and will cover all trial operations, including a HIPAA authorization for release of medical records, anthropometric data collection, performance of an abdominal exam, and collection of blood, urine, and stool biologic specimens, with the option for elective, de-identified specimen storage for future research.

### Additional consent provisions for collection and use of participant data and biological specimens {26b}

Collection of anthropometric and demographic data, performance of an abdominal exam, and collection of hematologic specimens by venipuncture will be conducted by a qualified study team member and stored in a de-identified manner as described above.

## Interventions

### Explanation for the choice of comparators {6b}

Participants will be randomized to either the experimental drug or placebo. An inert placebo is the appropriate comparator as this phase of research is to establish safety, and clinical efficacy is unknown. Should mechanistic data and/or participant reports suggest clinical benefit, future trials may include active comparators. The placebo contains 288 mg of rice protein, 109.3 mg microcrystalline cellulose, 4.3 mg Aerosil® 200 fumed silica, and 4.3 mg magnesium stearate encapsulated in an opaque orange gelatin capsule. Participants will be instructed to take one capsule per day, orally, with their first daily meal following randomization (Table 3). Bottles of placebo capsules will be stored in a locked cabinet prior to administration in climate-controlled conditions and monitored weekly for consistency in ambient temperature.

### Intervention description {11a}

The experimental drug will be 24 mg of 99%+ pure XN with 288 mg of rice protein, 109.3 mg microcrystalline cellulose, 4.3 mg Aerosil® 200 fumed silica, and 4.3 mg magnesium stearate encapsulated in an opaque orange gelatin capsule. Participants will be instructed to take one capsule per day, orally, with their first daily meal following randomization (Table 3). Bottles of the experimental drug will be stored in a locked cabinet prior to administration in climate-controlled conditions and monitored weekly for consistency in ambient temperature.

### Criteria for discontinuing or modifying allocated interventions {11b}

Participants may elect to withdraw from the trial at any time for any reason. Participants may be withdrawn by the study team if they experience any new-onset, moderate or severe adverse events from the intervention. Any a priori-specified critical lab value observed and confirmed on repeat analysis and attributable to the intervention may also result in participant withdrawal. The trial will be halted if >20% of all participants demonstrate laboratory abnormalities in per the criterion listed *or* experience adverse events of moderate or greater severity which are new from baseline or progressively worsening.

No modifications of intervention dose or frequency of administration are allowed, and participants will be considered non-adherent and withdrawn from the trial if deviations from the dosing standard are reported to the study team. As XN was demonstrated as safe and tolerable in healthy adults at the same dosage and length of time in our phase I study [15], and is considered Generally Recognized as Safe [20], it is not anticipated that serious harms will require early discontinuation of the product or medical intervention to address the issue.

### Strategies to improve adherence to interventions {11c}

Adherence will be monitored throughout the trial at weekly visits through verbal query and formally assessed at the week 4 and closeout visits by capsule count. Participants will be provided with sufficient study product for 5 weeks (35 capsules) at randomization and week 4 in anticipation of potential scheduling changes and/or unexpected losses. Participants will be considered adherent by taking >80% of expected doses throughout their participation in the trial. Participants will be provided suggestions to improve adherence such as daily phone alarms, consistent timing, reminder notes, etc. Returned study product will be stored in a locked cabinet in a locked room for future quality assessment.

### Relevant concomitant care permitted or prohibited during the trial {11d}

Allowable concomitant interventions are outlined according to the criteria in Table 2. Participants will be allowed rescue steroid medications for inflammatory flares associated with CD, although over-the-counter non-steroidal anti-inflammatory medications will be limited to no more than once per week.

### Provisions for post-trial care {30}

Participants will be contacted to determine the progression and resolution of any new-onset symptoms that appear following the baseline visit. The study team will send reports of any grade 3 or higher adverse events to the participant’s medical provider(s) following their participation in the trial.

### Outcomes {12}

The primary outcomes of the phase II trial include changes in laboratory toxicology measures, vital signs (blood pressure and heart rate), and AEs (Table 1). Assessment of laboratory measures to determine safety includes review for new-onset abnormalities (confirmed with repeat testing at the following clinical visit or as soon as possible following closeout, as applicable), including new-onset clinically significant elevation of liver function tests [aspartate transaminase (AST), alanine transaminase (ALT), bilirubin or alkaline phosphatase], new-onset anemia, elevations in creatinine, and/or reductions in estimated glomerular filtration rate (eGFR). Elevations of liver function tests will be considered “significant” if (1) abnormal per laboratory reference ranges and (2) represent a 25% deviation from previously observed values. New-onset anemia will be considered “significant” if (1) hemoglobin < 14 g/dL (140 g/L), hematocrit < 42% (< 0.42), or RBC < 4.5 million/mcL (< 4.5 × 10^12^/L) (for men); or hemoglobin < 12 g/dL (120 g/L), hematocrit < 37% (< 0.37), or RBC < 4 million/mcL (< 4 × 10 ^12^/L) (for women); *and* (2) values represent a 10% change or more from *baseline* values; *and* (3) values remain abnormal with repeat testing within 2 weeks. This threshold reduces reporting of new “borderline” abnormal values for participants with “high normal” baseline values. For elevations of creatinine, “significant” will be evaluated as greater than a 20% increase from *baseline* values, whereas reductions in eGFR will be considered as “significant” if greater than a 20% decrease from *baseline* values. A brief clinical interview will be conducted and recorded for all laboratory abnormalities not present at baseline to provide context, i.e., new-onset anemia tied to increased diarrheal episodes, etc. Serum calcium, potassium, and sodium; ɣ-glutamyl transferase (GGT); and blood urea nitrogen (BUN) to creatinine ratio (BUN:Cr) will also be collected to monitor electrolyte balance and provide additional measures of liver and kidney function, respectively.

The primary endpoint for laboratory values and vital sign measurements will be mean change from baseline to week 8, with changes also compared between groups from baseline to each additional follow-up visit (i.e., 2, 4, and 6 weeks). The frequency of new-onset or worsening laboratory values will be reported within groups throughout the trial at each biweekly visit. AEs and event severity will be reported as new-onset or worsening events spontaneously and at each biweekly visit. Together, these outcomes will provide a profile of safety and tolerability of 24mg XN for 8 weeks compared to placebo.

Secondary and exploratory outcomes include clinical disease activity, systemic inflammation (e.g., IL-10), intestinal permeability (e.g., intestinal fatty acid binding protein) and endotoxemia (e.g., LPS), platelet function, stool microbiome and bile acid composition, and the metabolic profile of XN in humans over 8 weeks. Each measurement will be collected at every biweekly clinical visit and compared between groups as mean change from baseline within groups and compared between groups at each follow-up visit. The primary endpoint for each secondary and exploratory outcome will be considered exploratory and reported as mean change within groups at week 8 from to baseline and compared between groups at each follow-up visit. The measurements to support these outcomes are outlined in Table 1.

### Participant timeline {13}

The flow of participant involvement and descriptions of study visit procedures are described in Fig. 1. Participants will be screened by phone to determine initial eligibility. Within 30 days of initial eligibility confirmation, participants will complete an in-person clinical screening visit to further confirm eligibility. If the candidate remains eligible, they will be invited to complete baseline and randomization visits within 30 days of screening. All additional visits will occur biweekly for 8 weeks and follow the procedure flow according to Fig. 1 and Table 3.

### Sample size {14}

A sample size of at least 12 participants per group provides 80% power to detect ≥50% difference in the proportions of participants experiencing laboratory abnormalities or adverse events at a threshold of *α* = .05 by *χ*^2^ test. This sample size, at *n* = 12 per arm, provides >90% power using 2-sided *t*-tests to detect a mean change of 0.25–0.5pg/mL in TNF-α in the treatment arm according to preliminary data from previous pilot studies. Accounting for attrition up to 25%, we will randomize up to 32 participants. Attrition during the phase I trial was 10% [15].

### Recruitment {15}

Participants will be recruited from the Portland, OR, area via word-of-mouth and via posted flyers at the institution’s clinic, local shops, and restaurants. Flyers will also be posted through online advertisements on a weekly basis. Indirect recruitment will occur through presentation at medical conferences at local support groups, such as the Northwest Chapter of the Crohn’s and Colitis Foundation, at which flyers will be available for attendees. Advertisements will also be placed through the Crohn’s and Colitis Foundation’s website and distributed through local chapter newsletters. The NUNM electronic health records system will be leveraged to generate a list of candidate participants based on ICD-9/10 codes with recruitment via email or direct mail. Oregon Health and Sciences University (OHSU) and its Oregon Clinical and Translational Research Institute (OCTRI) will be similarly leveraged to generate a list of candidate participants for direct recruitment.

## Assignment of interventions: allocation

### Sequence generation {16a}

The randomization sequence will be computer-generated and stratified according to biological sex to randomize 50% male and 50% female participants in each group.

### Concealment mechanism {16b}

A staff member of the Helfgott Research Institute uninvolved with the trial will maintain allocation concealment and create 32 sequentially numbered, opaque, and sealed envelopes containing the individual group assignments according to the randomization sequence.

### Implementation {16c}

Envelopes will remain maintained in a locked cabinet in a locked document room until the baseline visit when randomization occurs by one of the clinical investigators or coordinators. Thus, allocation will be concealed from study principal investigators (PI), coordinators, clinical investigators, participants, and statisticians until each participant is randomized.

## Assignment of interventions: blinding

### Who will be blinded {17a}

Blinding will be accomplished through multiple methods. Trained staff uninvolved in trial operations or analyses will produce a group code, receive and label product bottles accordingly, and generate stratified randomization sequences using freely available online software. To avoid any speculation about group allocation changes since the previous phase I trial, randomization group labels were altered to be “X2” and “Y1” (versus “A” and “B”). Upon randomization, participants will be assigned a new alpha-numeric ID reflecting their enrollment status. All parties involved in trial conduct will remain blinded until the primary trial analyses are deemed complete by all trial PIs; only then will group assignment be revealed. As secondary analyses are divided between collaborating sites, those directly involved with measurements and analyses will remain masked until these measures are completed and deemed final.

### Procedure for unblinding if needed {17b}

Unblinding will be permissible in the case of a serious adverse event requiring hospitalization or resulting in fatality, or if the data and safety monitoring board (DSMB) requires it upon regular review.

## Data collection and management

### Plans for assessment and collection of outcomes {18a}

Data collected from all participant interactions will be recorded on paper case report forms (CRFs) with electronic transcriptions stored in REDCap® [21, 22]. Physical data will be stored in a locked cabinet, in a locked document room, or in a building accessible only via badge or key. Electronic data will be stored in a de-identified manner and entered on a password-protected device by study staff into REDCap®. All data will be collected by study staff by verbal query or physical assessment other than the PROMIS-29 questionnaire which the patient will self-report directly into REDCap® on a password-protected tablet. All specimens will be labeled using a convention that includes participant ID, visit number, date stored, and specimen type.

Hematologic safety parameters in this study include those associated with liver function (AST, ALT, alkaline phosphatase, bilirubin, and GGT); kidney function (eGFR, BUN, creatinine, and BUN:Cr); general hematology (red blood cell count, white blood cell count, hematocrit, and hemoglobin); and electrolytes (sodium, potassium, calcium, and chloride). Spontaneous, self-reported, and serious adverse events will also constitute the safety and tolerability profile of XN. Retention, intervention adherence, and self-reported quality of life, measured through the Patient-Reported Outcomes Measurement Information System 29-item questionnaire (PROMIS-29) [23], will be considered measures of tolerability. All parameters will be collected at each visit and intervention adherence will be monitored at 4 weeks from baseline and at closeout.

Clinical activity will be evaluated using the Crohn’s Disease Activity Index (CDAI). The CDAI is a validated index based on clinical observations, laboratory values, and patient-reported outcomes [24, 25]. While there remains some controversy over the use of the CDAI, it correlates with other accepted indexes of clinical activity and some patient-reported subsets and further correlates with endoscopic activity indexes [26]. Abdominal examinations for masses will be performed by a licensed clinician.

Hematologic specimens will be collected by a trained phlebotomist or licensed clinician at the Helfgott Research Institute. Specimens used to establish safety parameters (CBC and CMP) will be analyzed through NW Quest Diagnostics® (Seattle, WA, USA). Hematologic specimens for serum and plasma analysis will simultaneously be collected and centrifuged at 1450 times *g* for 15 min within 2 h of collection. Plasma samples collected in lithium heparin-coated vacutainer tubes will be separated by serial 500-μL aliquots and stored in 1-mL graduated DNase-free, RNase-free, pyrogen-free cryovials (Simport, Beloeil, Quebec) at −70°C at the study site. Circulating concentrations of inflammatory biomarkers, including tumor necrosis factor-α and interleukins (e.g., IL-1, 10, 12, and 17); intestinal permeability markers (e.g., CD14 and intestinal fatty acid binding protein); and markers of endotoxemia (i.e., lipopolysaccharide and its binding protein) will be assessed by flow cytometry-based multiplex assays [27], enzyme-linked immunosorbent assay (ELISA) [28], and Limulus amebocyte lysate assay [29, 30], respectively.

Sodium citrate-anticoagulated blood samples (20 mL/participant) will be processed within 2 h of collection for platelet studies at baseline, week 4, and closeout. A small volume of whole blood (50 μL) from each sample will be prepared for flow cytometry analysis as previously described [31, 32]. For biochemical studies, platelets will be purified from whole blood samples by centrifugation and resuspended in a modified HEPES/Tyrode (H-T) buffer as previously described [32]. Washed platelet samples will be treated prior to lysis, flash frozen, and stored at −80°C. Subsequent analyses will measure specific biochemical and intracellular signaling responses to assess the effect of XN on platelet function using Western blot and by quantitative mass spectrometry, as previously described [32].

All stool samples are expected to be collected the morning of each visit from a single bowel movement; however, in instances where participants collect stool samples the night before their visit, they will be instructed to place all samples in a residential freezer prior to transportation to the research facility to preserve specimen integrity. Fecal calprotectin (FCP) will be quantified using the Calprotectin ELISA Assay kit (Epitope Diagnostics, Inc., San Diego, CA, USA). Gut microbial and bile acid compositions will be determined from the OmniGene®GUT Kit (DNA Genotek, Ottawa, ON, Canada) and subsequent procedures as described in detail by partner sites [33, 34].

Analytes of XN will be collected at each visit beginning at baseline via circulating plasma, a 24-h urine collection, and stool samples to establish a longitudinal profile of metabolism and isomerization. Identification and quantification of XN and its metabolites, including isoxanthohumol, 6-prenylnaringenin, 8-prenylnaringenin, O-desmethylxanthohumol, dihydro-*O*-desmethylxanthohumol, and α,β-dihydroxanthohumol, will be achieved by using ultra-high-performance liquid chromatography-triple quadrupole time-of-flight mass spectrometry (LC-MS/MS) as previously described [35, 36]. Plasma samples, and stool samples without fixatives, will be collected and stored according to the processes outlined above. Urine specimens will be separated from the 24-h sample following homogenization in serial 1-mL aliquots to be stored in 1-mL graduated DNase-free, RNase-free, pyrogen-free cryovials (Simport, Beloeil, Quebec) at −70°C at the study site.

### Plans to promote participant retention and complete follow-up {18b}

Participants will be provided a minimum of one reminder email and/or phone call prior to each visit. Retention strategies include stipends upon completion of the clinical screening visit ($50) and completion of subsequent follow-up visits except the randomization visit ($190), for up to a total of $1000. In case of campus closure wherein participants will be required to travel to off-site phlebotomy clinics, additional compensation up to $200 may be provided. Finally, participants may be reimbursed for travel and/or lodging expenses if travel is required for participation.

### Data management {19}

To minimize human data entry errors, all data entered in REDCap® will be marked “unverified” for a member of the study team to later confirm the accuracy of the data entry, at which point will be changed to “complete.” Only data marked “complete” will be considered for analysis. Authorization for data exports will be limited to the study PI, clinical investigators, and biostatistician. All data will be kept for a minimum of 6 years following completion of all grant-associated activities.

### Confidentiality {27}

All data shared between institutions will follow procedures outlined in a signed data use agreement and uploaded to secure portals for confidentiality and privacy protection. All data stored in an electronic manner will be de-identified using alpha-numeric codes. Physical files which may include identifiable information will be stored in a key-locked cabinet inside a key-locked room within a keycard-locked and alarm-coded building. Only employees of the institution may be granted keycard access to the building and storage room while only clinical investigators and study coordinators will have access to the keys to the cabinets.

### Biological specimens for future use {33}

Participants will have the opportunity to consent for additional aliquots of urine and plasma to be collected and maintained for future use during the baseline visit and will be requested to provide verbal confirmation or withdrawal of consent at follow-up visits. These specimens will include up to four aliquots of 500 μL plasma and 1mL urine each for potential future exploratory analysis. Samples will be similarly de-identified and stored using the mechanisms above. No genetic analysis will be conducted.

## Statistical methods

### Statistical method outcomes {20a}

Laboratory measures will be analyzed first with descriptive statistics including mean, median, and standard deviation. Distributions will be tested for skew and transformed (e.g., natural log) as needed to reduce skew on subsequent analyses. If distributions indicate substantial non-normality that cannot be modified with transformation, non-parametric tests (e.g., Wilcoxon rank-sum) will be used.

In addition, the frequency of new, clinically significant laboratory abnormal values as outlined above will be monitored by the DSMB. The percentage of these new abnormal values per reference ranges will be reported at each time point and compared between groups by Fisher’s exact test. Assessment for changes in the distribution of each parameter will be presented as means and confidence intervals (CI) within and between groups. The significance of differences will be compared using 2-sided, unpaired *t*-tests if normally distributed and a non-parametric Wilcoxon rank-sum test of mean values between groups if non-normally distributed. Overall increases and decreases in laboratory parameters will be tested using linear mixed ANOVA with time point as a repeated factor, potentially suggestive of cumulative toxicity if trending in a clinically deleterious direction. All factors will be considered significant if *p* < .05.

Clinical activity will be measured through the CDAI. A score >150 is considered *clinically active* and is an eligibility criterion. Changes in score from baseline between groups will be compared within groups using two-sided, unpaired *t*-tests at each interval with the primary outcome as a change in value from baseline to closeout, whereas within-group changes will be compared using two-sided, paired *t*-tests. Non-parametric Wilcoxon rank-sum test of mean values between groups will be used if data are non-normally distributed.

Measures within the PROMIS-29 v.2.0 questionnaire include seven validated subscales, i.e., physical function, anxiety, depression, pain intensity, pain interference, sleep disturbance, and social interaction. Analyses will be performed by calculating descriptive statistics of mean, median, and mode of reported *T*-scores and overall PROMIS-29 questionnaire and each subscale. Welch’s *t*-test will be used to test the differences in scores between groups with *p* < .05 considered significant.

As this study will have a relatively small sample size, changes in secondary and tertiary outcome values will be considered preliminary but will be compared to results found in the phase I trial. Assessments for these outcomes, including inflammatory cytokines, gut permeability biomarkers, markers of endotoxemia, bile acids, and platelet function, will be calculated using Cohen’s *d* statistic for effect size and qualitatively compared for differences between trials using changes in values between baseline and closeout. The primary effect estimate will be for changes between baseline and closeout. This study is powered to detect a difference in Cohen’s *d* value of at least 1.2 between groups. Results will be considered evidence of a possible effect if a Cohen’s *d* value > 0.5 is observed. Statistical comparisons for significance will be considered exploratory and will be limited to unpaired *t*-tests between XN treatment groups.

The metabolite profiles and impact of XN on gut microbial composition and platelet function will be conducted with specific methods previously described by partner sites [31, 33]. Correlations between changes in CDAI and microbiota changes, inflammatory biomarker changes, and/or bile acid changes will be considered exploratory.

### Interim analyses {21b}

Statistical interim analyses will not be conducted; however, regular meetings of the study staff, DSMB, and auditing of the trial by third parties will be conducted as described below.

### Methods for additional analyses {20b}

Currently, no subgroup analyses are planned. If significant differences in CDAI scores are observed between groups at baseline, ANCOVA will be performed for all tests of mean differences (e.g., circulating inflammatory cytokines), adjusting for baseline CDAI scores between groups.

### Methods in analysis to handle protocol non-adherence and any statistical methods to handle missing data {20c}

Given the safety-oriented and mechanistic nature of the trial, all analyses will be conducted per protocol; however, as a sensitivity analysis, missing laboratory data will be imputed by multiple imputation to assess the robustness of study results.

### Plans to give access to the full protocol, participant-level data, and statistical code {31c}

There are no plans for granting public access to the full protocol, participant-level dataset, or statistical code.

## Oversight and monitoring

### Composition of the coordinating center and trial steering committee {5d}

Clinical investigators, study coordinators, and the clinical site PI will meet on a weekly basis to discuss ongoing business associated with the trial. Amendments to the trial protocol will be sent to the appropriate agency [NCCIH, US FDA, and/or the NUNM Institutional Review Board (IRB)] according to their required timeline. Annual reviews of the trial’s protocol and progress will occur through the NUNM IRB. All study team members will meet regularly to discuss trial developments and administration. The study coordinator will maintain minutes of these meetings, which may be brandished upon request of the DSMB or third-party auditor.

### Composition of the data monitoring committee, its role, and reporting structure {21a}

The DSMB will be composed of a medical doctor, a doctor of osteopathy, a biostatistician, and a toxicologist. The DSMB will review regular reports produced by the study team to ensure safe and responsible conduct of the trial and make any determination of discontinuation. Final reports from the DSMB will be reviewed by the NUNM IRB during annual continuing reviews for approval renewal of trial operations.

### Adverse event reporting and harms {22}

Adverse events will be assessed and graded according to severity at each biweekly clinical visit beginning at the baseline visit, as well as accepted via spontaneous report. Self-reported adverse events will be administered by verbal query through a standardized multi-system questionnaire based on the National Cancer Institute’s Common Technology Criteria for Adverse Events v4.0. Adverse events reported between study visits will be termed “spontaneous adverse events” and graded in a similar manner according to self-reported adverse events according to severity and duration. If an adverse event is of moderate or greater severity, the study team will coordinate with the patient’s physician and develop and document a response or monitoring plan.

Adverse events, both self-reported and spontaneous, will be detailed according to severity and length of duration and compared between groups. The presence of symptoms at baseline will inform the attributability of any symptom to the intervention. Worsening or new-onset symptoms after baseline will be considered potentially attributable to the intervention and will be determined according to prevalence in each group and a case-by-case basis by the study team and DSMB.

### Frequency and plans for auditing trial conduct {23}

Third-party audits and regular DSMB meetings will occur at half-enrollment (initiated after at least 12 participants but no more than 17 have completed the trial) and upon completion of the trial, or per requirements of the NCCIH.

### Plans for communicating important protocol amendments to relevant parties {25}

Potential modification to the protocol, study staff, or trial operations will be submitted to the NUNM IRB for review and approval. Upon approval, any major modifications to eligibility criteria will be submitted to NCCIH and the FDA for review and approval. Upon approval by these agencies, any trial registries will be subsequently updated reflecting these changes.

### Dissemination plans {31a}

Trial results will be shared to participants upon publication of any relevant manuscripts. Publication of safety and tolerability findings and secondary outcomes may be published separately. Additionally, patient advocacy and support groups such as the Crohn’s and Colitis Foundation will be contacted to provide synopsized findings and publications upon request.

## Discussion

In this article, we present the protocol for a phase II trial designed to measure the safety and tolerability profile of XN over an 8-week period in adults with clinically active CD. XN has demonstrated regulatory effects on intestinal permeability via FXR [12, 13], reduction of inflammatory cytokine production through inhibitory actions on NFκB and modulation of FXR and NRF2 [8–13], and modulation of the gut microbiome through its prebiotic activity in non-human models [6, 7]. Thus, there is promise for XN to positively impact clinical activity in CD in a safe manner via multiple relevant mechanistic pathways. This trial will determine whether these mechanisms of XN translate to humans based on the clinical activity of CD, inflammatory biomarkers, intestinal permeability, and gut microbial composition. Furthermore, flavonoids have demonstrated modulatory activities relating to platelet function [37]. Therefore, exploring such potential in CD may guide therapeutic considerations in its preventing long-term risk of thrombotic events [38].

Strengths of this trial protocol include its design and the meticulous efforts to ensure data accuracy, participant retention, and safety. The ability to perform direct comparisons of results from the preceding trial in healthy adults supports higher impact and validity of findings in this study. Furthermore, this research is a strong example of inter-disciplinary and cross-institutional collaboration, adding rigor and innovative methods to the study of a natural drug candidate.

We have observed several limitations in the current protocol since commencing this trial. First, the demographic distribution of the Portland, OR metropolitan area is predominantly non-Hispanic white and will limit the generalizability of findings from this trial. Second, the study has faced several challenges that limit recruitment efforts. For example, the population in the Portland area has high usage of several forms of anti-inflammatory compounds prohibited in this study (e.g., *Cannabis* products and turmeric). The COVID-19 pandemic has compounded these challenges for multiple reasons: quarantine regulations encumbered recruitment efforts, national vaccine administration posed restrictions on participant eligibility, and participants with chronic conditions face a disproportionate threat from infection [39]. Furthermore, CD has a relatively low prevalence in the general population which can challenge recruitment penetration. Additionally, the use of a 150-point CDAI cutoff as a tool for determining clinical activity further restricts participant eligibility. Objectively defining “active” disease in this way may dismiss individual participant experience of chronic disease. Finally, this trial is limited by the lack of dietary pattern data, which has the potential to confound data associated with the impacts of XN on the gut microbiome [40].

Nevertheless, this phase II trial will generate a substantial source of data for direct comparison to an otherwise healthy population in the phase I trial. Rigorous investigations into the safety and tolerability of natural drug candidates like XN will support and inform similar trials in the future. While the findings of this trial will be of great interest to clinical translational investigators and healthcare providers, it will also be impactful for patients seeking safe, adjunctive treatments for CD or other chronic inflammatory conditions affected by gut permeability such as rheumatoid arthritis [41, 42].

## Trial status

IRB #RB71720. Approval date: July 17, 2020. Current protocol version: v1.13. Recruitment start date: August 1, 2020. Projected recruitment end date: December 31, 2022. Current enrollment as of April 28, 2022: 15 participants completed of up to 32.

## Data Availability

The data generated by this study will be available upon request to the corresponding author.

## Abbreviations

NCCIH: National Center for Complementary and Integrative Health
NHLBI: National Heart, Lung, and Blood Institute
XN: Xanthohumol
Nrf2: Nuclear erythroid 2-related factor 2
NFκB: Nuclear factor-κ B
FXR: Farnesoid-X receptor
CD: Crohn’s disease
CDAI: Crohn’s Disease Activity Index
FDA: The United States Food and Drug Administration
AST: Aspartate aminotransferase
ALT: Alanine aminotransferase
GGT: ɣ-Glutamyl transferase
eGFR: Estimated glomerular filtration rate
BUN: Blood urea nitrogen
Cr: Creatinine
PROMIS-29: Patient-Reported Outcomes Measurement Information System 29-item questionnaire
FCP: Fecal calprotectin
IND: Investigational new drug
CBC: Complete blood count
CMP: Comprehensive metabolic panel
OHSU: Oregon Health and Sciences University
OCTRI: Oregon Clinical and Translational Research Institute
PI: Principal investigator
DSMB: Data and Safety Monitoring Board
CRFs: Case report forms
CI: Confidence interval
IRB: Institutional review board
NUNM: National University of Natural Medicine
